# Bridging Haemato-Oncology and Critical Care: Survival and Functional Outcomes From a Portuguese Central Hospital

**DOI:** 10.7759/cureus.98135

**Published:** 2025-11-30

**Authors:** Diana Martins Fernandes, André Airosa Pardal, Paula Ferraz, Rita Queirós Pereira, Marisa Simões, Marta Henriques, Francisco Dias, André Valois, Patrícia Rocha Silva, Lúcia Meireles Brandão, Guiomar Castro, Marta Couto, José Artur Paiva, Nuno Príncipe

**Affiliations:** 1 Intensive Care Medicine, Unidade Local de Saúde de São João, Porto, PRT; 2 Haemato-Oncology, Unidade Local de Saúde de São João, Porto, PRT; 3 Pharmacology, Unidade Local de Saúde de São João, Porto, PRT; 4 Immunotherapy, Unidade Local de Saúde de São João, Porto, PRT; 5 Intensive Care Medicine, Unidade Local de Saúde de Matosinhos, Porto, PRT

**Keywords:** haematology malignancy, haemato-oncology, intensive care unit, performance status, survival

## Abstract

Background

Despite substantial therapeutic progress in haemato-oncology (HO), referring exclusively to patients with haematological malignancies, ICU admission decisions for these patients are often hindered by the perception of poor prognosis. This study aimed to evaluate the one-year survival and functional outcomes of critically ill HO patients following hospital discharge.

Methods

A retrospective, single-centre observational study was conducted in a Portuguese tertiary care centre, including 54 consecutive non-elective ICU admissions of adult HO patients between January 2022 and October 2023. The primary endpoint was one-year survival post-hospital discharge, while secondary endpoints included maintenance of treatment plans and functional recovery assessed using the Eastern Cooperative Oncology Group (ECOG) performance status one year after hospital discharge.

Results

Seventy-four percent of patients survived their ICU stay, with survival rates of 61% at six months and 50% at one year post-discharge. Among one-year survivors, 93% exhibited good performance status (ECOG 0-1) and continued their intended oncological treatment. Only 7% transitioned to palliative care. Despite a high burden of organ dysfunction (87% with multiple dysfunctions) and significant therapeutic needs, including mechanical ventilation (33%) and renal replacement therapy (50%), functional recovery and treatment resumption were achieved in most survivors.

Conclusions

Critically ill HO patients can achieve meaningful survival and functional recovery following ICU admission. The findings challenge prior biases against ICU admissions for this population and underscore the need for early, multidisciplinary intervention to optimize outcomes.

## Introduction

Over the last two decades, Haemato-Oncology (HO) patients, referring exclusively to those with haematological malignancies, have benefited considerably from therapeutic advances, leading to improved survival [1-2]. As a result, a growing number of HO patients are at risk of life-threatening acute illness driven by infection, disease-specific emergencies, treatment toxicity, and decompensation of comorbid conditions, and will increasingly require ICU admission [1-2]. Nonetheless, referring HO patients for critical care remains challenging, as the perception of poor outcomes has traditionally biased decision-making [3].

Identifying the best candidates and timing for ICU admission remains a complex challenge. HO patients’ clinical characteristics are of paramount importance and often distinct from those with solid tumours. Studies have shed light on this issue, and classic mortality predictors have lost their value. Factors such as the haematological disease itself, neutropenia, or a history of haematopoietic stem-cell transplant (HSCT) are no longer associated with worse outcomes [3-4]. Beyond necessary changes in admission policies, critically ill HO patients have also been shown to benefit from extended intensive care trials, more so than other cancer populations [5].

To optimize outcomes, ICU teams should focus on high-value care. This includes new triage criteria for ICU admission, early ICU admission upon identification of organ dysfunction, tailored management of respiratory failure and neutropenic sepsis, avoidance of high-risk procedures, and close multidisciplinary collaboration incorporating cancer specialists [6]. In contemporary Haematology, ICU admission is not a last resort, detached from the patient’s therapeutic plan, but rather a key treatment element and a bridge toward survival and effective disease management [7]. On the other hand, HO patients may pose a substantial psychological burden on ICU teams, as therapeutic obstinacy and end-of-life discussions arise frequently and are common sources of conflict within critical care settings [8].

Given the challenges often faced by HO patients in accessing intensive care and the limited understanding of their functional outcomes after ICU discharge, it is essential to determine whether recent advances in critical care have improved survival rates in this population and how these advances affect functional recovery. Bearing this in mind, the present study aims to characterize survival, maintenance of treatment plans, and functional recovery of HO patients admitted to the ICU one year after hospital discharge.

## Materials and methods

Study design and patients

A retrospective observational study was conducted on consecutive non-elective HO patients admitted to the ICU at a tertiary university hospital centre. Inclusion criteria encompassed adults aged 18 years or older admitted to the ICU between January 2022 and October 2023, with either an active haematological malignancy, a haematological malignancy in remission for less than five years, or HSCT within the past five years. Exclusion criteria included complete remission of the malignancy for more than five years, ICU admission solely to maximise the safety of a procedure, and patients aged 18 years or younger. No patients with solid tumours were included.

Endpoints and statistical analysis

The primary outcome was survival at one-year follow-up. Secondary outcomes included maintenance of the intention-to-treat plan and performance status, as measured by the ECOG scale, with good performance status defined as ECOG 0 or 1. Included patients were followed throughout their entire hospital stay and for one year after hospital discharge. Outcomes were evaluated at hospital discharge and at 6 and 12 months after discharge. For statistical analysis, SPSS (IBM Corp., released 2022; IBM SPSS Statistics for Windows, version 29.0, Armonk, NY, USA) was used. Data were presented as n (%) or reported using mean and SD or median and IQR, including the full range of distribution (minimum and maximum). Normality assumptions were assessed with the Shapiro-Wilk and Kolmogorov-Smirnov tests.

Data collection

Data collection included patients’ demographics, performance status, past medical history, haemato-oncological diagnosis and treatment-related variables, intensive care-related treatments, and outcomes, including survival, cancer treatment plan, and performance status. Performance status was classified using the Eastern Cooperative Oncology Group (ECOG) score [9]; a value for individual performance status was assigned for premorbid functional status and reassessed at one-year follow-up after hospital discharge. The burden of chronic comorbidities was assessed using the Charlson Comorbidity Index determined at ICU admission. ICU readmission was considered if it occurred within 48 hours of discharge. Reasons for ICU admission were recorded based on the main syndromes present at ICU admission. Acute respiratory failure was defined as oxygen saturation less than 90% or PaO₂ less than 60 mmHg on room air combined with dyspnoea, a respiratory rate greater than 30 breaths per minute, or clinical signs of respiratory distress. Sepsis was defined as life-threatening organ dysfunction, identified as an acute change in total Sequential Organ Failure Assessment (SOFA) score ≥2 points consequent to infection [10]. Shock was defined as persistent hypotension requiring vasopressors to maintain mean arterial pressure ≥65 mmHg and a serum lactate level >2 mmol/L despite adequate volume resuscitation [10]. Infections were considered nosocomial if acquired in the hospital setting, occurring ≥48 hours after admission to the healthcare facility [10]. Neutropenia was defined as a neutrophil count of less than 1500/mm³, and severe neutropenia as less than 500/mm³. Severity of illness was measured with the SOFA, Acute Physiology and Chronic Health Evaluation (APACHE) II, and Simplified Acute Physiology Score during the first 24 hours of ICU admission. Haematological prognosis of the main patient groups was based on recognised prognostic frameworks: acute myeloid leukaemia was staged according to the 2022 European LeukaemiaNet risk stratification [11]; the Durie-Salmon staging system was used for multiple myeloma; the International Prognostic Scoring System (IPSS) for myelodysplastic syndromes (MDS); and Ann Arbor staging for lymphomas.

This study was conducted under the framework of the Strengthening the Reporting of Observational Studies in Epidemiology (STROBE) 2014 guideline [12]. The collected data and follow-up to assess one-year survival, ECOG performance status, and treatment continuation were obtained retrospectively through electronic medical records. The Institutional Ethics Committee reviewed and approved the study and waived the requirement for patient consent (Approval number: CES 381/2024). All procedures followed ethical standards and complied with the Declaration of Helsinki. Data processing was conducted anonymously, adhering to the principles outlined in the European General Data Protection Regulation (GDPR).

Setting and admission procedures 

The centre operates a 60-bed Intensive Care Department, which includes a 10-bed ICU dedicated to immunocompromised patients, including haematological patients. Patients are reviewed by an intensivist team, with at least one intensivist available 24 hours per day, and supported by a senior Haematology specialist, also available 24 hours a day. Early referral from the haematology ward is standard practice. ICU admission decisions are made jointly by haematologists and intensivists, based on clinical judgement and perceived reversibility of organ dysfunction, as there are no local standardised admission criteria for HO patients in our institution. The ICU has access to all medical specialties and resources available in the centre, including pharmacists, nutritionists, rehabilitation specialists, psychological counselling, and palliative care specialists, who work based on referrals. Life-supporting interventions, anti-infective agents, prophylactic treatments, and diagnostic procedures were administered at the discretion of the attending intensivists, in line with best clinical practice and guidelines. Chemotherapy, corticosteroids, haematopoietic growth factors, immunosuppressive drugs, and other cancer-related treatments were prescribed by the senior haematologist responsible for each patient, in accordance with international guidelines.

The nurse-to-patient ratio in the ICU is 1:2 in the open area and 1:1 in isolation rooms. The unit is equipped with four high-efficiency particulate air (HEPA)-filtered single isolation rooms designed for patients with severe neutropenia or those undergoing stem-cell transplants to prevent infections, and these rooms operate under a strict hygiene protocol (hand hygiene, specialised clothing, and limited visitor access).

## Results

Patient characteristics

A total of 54 patients with haematological malignancies were admitted to the ICU during the study period. Patient characteristics at ICU admission are shown in Table 1.

**Table 1:** Patient characteristics. ECOG PS: Eastern Cooperative Oncology Group performance status; APACHE II: Acute Physiology and Chronic Health Evaluation II; SAPS II: Simplified Acute Physiology Score II; SOFA: Sequential Organ Failure Assessment.

Variable	Value
Age, median (P25-P75); min-max	55.5 (39-64); 22-76
Sex, % (n)	
Male	52% (n = 28)
Female	48% (n = 26)
ECOG performance status, % (n)	
ECOG 0-1	81% (n = 44)
ECOG 2-4	19% (n = 10)
Charlson Comorbidity Index, median (P25-P75)	3 (2-4)
Comorbidities, % (n)	
Chronic obstructive pulmonary disease	24.1% (n = 13)
Arterial hypertension	22.2% (n = 12)
Diabetes mellitus	22.2% (n = 12)
Heart failure	14.8% (n = 8)
Chronic kidney disease	9.2% (n = 5)
Admission source, % (n)	
Hospital ward	89% (n = 48)
Operating room	6% (n = 3)
ER	4% (n = 2)
Other hospital	2% (n = 1)
Reason for ICU admission, % (n)	
Sepsis/septic shock	51.9% (n = 28)
Acute respiratory failure	18.5% (n = 10)
Haemato-oncology emergencies	13% (n = 7)
Other	16.7% (n = 9)
APACHE II, mean ± SD	25.4 ± 6.7
SAPS II, mean ± SD	53.3 ± 17.6
SOFA, mean ± SD	8.22 ± 3.1
Days from hospital to ICU admission, median (P25-P75); min-max	15.5 (6-18); 0-133
One or more referrals before ICU admission, % (n)	24% (n = 13)

As listed in Table 2, the most prevalent underlying haematological malignancies were AML followed by multiple myeloma, mostly de novo diagnoses, and 28% were in a relapsed/refractory state. The median time from haematological malignancy diagnosis to ICU admission was 106 days (P25-P75 30-365), with 21 patients (38.9%) diagnosed within the previous three months. Approximately 78% of patients received chemotherapy in the 30 days prior to ICU admission, 62% (n = 26) of whom were receiving first-line chemotherapy. The median time from the last dose of chemotherapy to ICU admission was 4.2 days (P25-P75 2-12.75). Only one patient was admitted after response to third-line chemotherapy for relapsed chronic myeloid leukaemia.

**Table 2:** Patient malignancy description according to the 5th WHO classification. Risk stratification was pathology-specific: for AML, we used the European LeukaemiaNet (ELN) 2022 guidelines; Durie-Salmon staging for multiple myeloma; and Ann Arbor staging for adult cell lymphomas. AML: Acute myeloid leukaemia.

Underlying haematological malignancy and staging	
Disease status at admission, % (n)	
Newly diagnosed	72.2% (n = 39)
Relapsed/refractory disease	27.8% (n = 15)
Days since diagnosis, median (P25-P75); min-max	106 (30-365); 0-423
Acute myeloid leukaemia, % (n)	55.5% (n = 30)
Intermediate	33.3% (n = 18)
Favourable	14.8% (n = 8)
Unfavourable	5.6% (n = 3)
Acute promyelocytic leukaemia, % (n)	1.9% (n = 1)
Multiple myeloma, % (n)	18.5% (n = 10)
II-A	5.6% (n = 3)
III-A	5.6% (n = 3)
III-B	7.4% (n = 4)
Diffuse large B-cell lymphoma, not otherwise specified, % (n)	5.6% (n = 3)
Stage II B	1.9% (n = 1)
Stage IV B	3.7% (n = 2)
B-lymphoblastic leukaemia, not otherwise specified, % (n)	5.6% (n = 3)
Myelodysplastic neoplasms, % (n)	3.7% (n = 2)
Intermediate-risk	1.9% (n = 1)
High-risk	1.9% (n = 1)
Adult T-cell lymphoma, % (n)	3.7% (n = 2)
Stage IV	3.7% (n = 2)
Mixed lineage leukaemia, % (n)	1.9% (n = 1)
T-prolymphocytic leukaemia, % (n)	1.9% (n = 1)
T-lymphoblastic leukaemia, not otherwise specified, % (n)	1.9% (n = 1)
Chronic myeloid leukaemia, % (n)	1.9% (n = 1)
Chemotherapy before ICU admission, % (n)	78% (n = 42)
First-line	62% (n = 26)
Second-line	36% (n = 15)
Third-line	2% (n = 1)
Last day of chemotherapy, median (P25-P75); min-max (days)	4.2 (2-12.75); 0-133
Haematopoietic stem-cell transplant recipient, % (n)	
Autologous	16.7% (n = 9)
Allogeneic	16.7% (n = 9)

Haematopoietic stem-cell transplantation was performed in 18 patients (29.6%): autologous in 9 (16.7%) and allogeneic in 9 (16.7%). The majority of patients were admitted from the HO ward. The median time between hospital admission and ICU admission was 15.5 days (P25-P75 6-18; range 0-133). Only five patients (9%) were admitted to the ICU within one day of hospital admission; two of these (4%) were direct admissions from the ED, one with AML and the other with multiple myeloma. The main reasons for ICU admission were sepsis/septic shock, followed by acute respiratory failure. The mean APACHE II and SAPS II scores were 25.4 (±6.7) and 53.3 (±17.6), respectively, and the mean SOFA score was 8.22 (±3.1), indicating moderate-to-severe organ dysfunction on admission. Regarding triage decisions, 24% (n = 13) of patients required more than one referral from the haematologist before ICU admission; the main reason for initial refusal was the presence of only a single organ dysfunction, deemed insufficient to warrant ICU admission.

ICU management and life-supporting interventions

Variables related to ICU management and the use of life-supporting interventions are summarised in Table 3. In this cohort, 87% (n = 47) experienced two or more organ dysfunctions and 78% (n = 42) three or more. Respiratory, renal, and cardiovascular dysfunction were the most frequent.

**Table 3:** Admission-related variables and organ support in the ICU. PaO₂: Partial pressure of arterial oxygen; FiO₂: Fraction of inspired oxygen.

ICU-related variables and organ support	
Acute respiratory failure, % (n)	90.7% (n = 49)
PaO₂/FiO₂, median (P25-P75)	212 (199-473)
Aetiology of acute respiratory failure, % (n)	90.7% (n = 49)
Respiratory sepsis	30.6% (n = 15)
Non-respiratory sepsis	28.6% (n = 14)
Hypervolaemia	10.2% (n = 5)
Leukostasis	4.1% (n = 2)
Diffuse alveolar haemorrhage	6.1% (n = 3)
Undetermined	20.4% (n = 10)
Respiratory support, % (n)	
High-flow oxygen	44.4% (n = 24)
Non-invasive ventilation	55.0% (n = 27)
Invasive mechanical ventilation	33.3% (n = 18)
Extracorporeal membrane oxygenation	5.6% (n = 3)
Time to intubation, days, mean ± SD	2.0 ± 2.7
Duration of invasive mechanical ventilation, days, mean ± SD	18.1 ± 18.6
Complete blood count variables, median (P25-P75)	
Haemoglobin	8.1 (7.0-8.9)
Neutrophil count	0 (0-945)
Platelet count	12 (10-23.75)
Blood products transfused, median (P25-P75)	
Red-blood-cell transfusions	5 (2-9.8)
Platelet transfusions	8 (3-11)
Acute kidney injury, % (n)	74.0% (n = 40)
Renal replacement therapy, % (n)	50.0% (n = 20)
Vasopressors, % (n)	61.0% (n = 33)
Admission lactate, median (P25-P75)	2.63 (1.29-4.00)
Chemotherapy in the ICU, % (n)	14.8% (n = 8)
Corticosteroids, % (n)	46.3% (n = 25)
Herpes simplex virus prophylaxis, % (n)	94.4% (n = 51)
Pneumocystis jirovecii prophylaxis, % (n)	20.4% (n = 11)

Regarding laboratory work-up and immunosuppression risk, either related to the underlying malignancy or its treatment, 76% exhibited neutropenia and 63% severe neutropenia, with a median absolute neutrophil count of 0/µL (P25-P75 0-945). None of the patients received antibiotic prophylaxis, but 94% received antiviral prophylaxis against herpetic infections and 20% were on prophylaxis for Pneumocystis pneumonia. Sepsis and septic shock remained the leading causes of ICU admission, with a predominance of a respiratory focus and nosocomial aetiology (83%). Thirty-seven patients (69%) had a temporary central venous catheter in place; 7 (13%) had long-term tunnelled central venous catheters.

Acute respiratory failure was the second leading cause of admission, with a median PaO₂/FiO₂ ratio of 212 (P25-P75 199-473). Pneumonia accounted for 31% (n = 15) of cases and was predominantly nosocomial. Despite exhaustive investigation, including thoracic CT and bronchofibroscopy with bronchoalveolar lavage, the aetiology remained undetermined in 20% (n = 10) of patients. Intubation and invasive mechanical ventilation were required in 33% (n = 18), with a median time from ICU admission to intubation of 1 day (P25-P75 0-2.5; range 0-9) and a median ventilation duration of 11.5 days (P25-P75 6.5-21.8; range 1-63). Three patients were rescued with primary awake extracorporeal membrane oxygenation after failure of non-invasive respiratory support.

Acute kidney injury occurred in 40 patients (74%), most frequently among those with AML (n = 30, 77%) and plasma-cell dyscrasias (n = 10, 19%). Sepsis, hypoperfusion, and nephrotoxic agents were the main aetiological factors. Renal replacement therapy was required in 20 patients (50%), for a median duration of 7.5 days (P25-P75 4.3-11.8; range 2-66). Anticoagulation (regional or systemic) was regularly avoided because of profound thrombocytopenia in most patients, with a median platelet count of 12/µL (P25-P75 10-23.75), conferring a high haemorrhagic risk.

Vasoactive support was required by 61% of patients (n = 33), with noradrenaline being the most commonly administered agent. About 24% (n = 13) required two or more vasoactive agents. The median admission lactate level was 2.63 mmol/L (P25-P75 1.29-4.00; range 0.70-19.57).

At ICU admission, nearly half of the patients (n = 26, 48%) had a haemoglobin concentration below 8 g/dL, with a median of 8.1 g/dL (P25-P75 7.0-8.9). The majority (n = 48; 89%) required at least one RBC transfusion, with a median of 5 transfusions per patient (P25-P75 2-9.8). Thrombocytopenia was observed in nearly all patients (98%) on admission, with a median platelet count of 12/µL (P25-P75 10-23.75), indicating severe thrombocytopenia. A median of 8 platelet transfusions per patient (P25-P75 3-11) was documented.

Regarding haematological treatments, chemotherapy was administered at some point during the ICU stay in 15% (n = 8), primarily for newly diagnosed multiple myeloma or AML in patients who had not previously received chemotherapy. No chemotherapy-related adverse events were documented during the hospital stay. Four patients with AML underwent leukapheresis for the management of hyperleukocytosis. Corticosteroid therapy was given to 46% (n = 25), with 15% (n = 8) receiving it as part of their haematological malignancy treatment. Granulocyte-colony stimulating factors were prescribed for 12 patients (22%) at some point during their ICU stay.

Survival and performance status

Patient outcomes are listed in Table 4. The median ICU length of stay was 8 days (P25-P75 3-15; range 0-79), with no readmissions documented within 48 hours of ICU discharge. The median hospital length of stay was 40 days (P25-P75 29-50; range 5-174).

**Table 4:** Patient outcomes. ECOG PS: Eastern Cooperative Oncology Group performance status.

Patient outcomes	
Length of ICU stay, days, median (P25-P75)	8 (3-15)
Length of hospital stay, days, median (P25-P75)	40 (29-50)
Survival at ICU discharge, % (n)	74.1% (n = 40)
6-month survival, % (n)	61.1% (n = 33)
1-year survival, % (n)	50.0% (n = 27)
1-year performance status, % (n)	
ECOG 0-1	92.6% (n = 25)
ECOG 2-4	7.4% (n = 2)
1-year haematological treatment plan, % (n)	
Curative-intent treatment / surveillance	92.6% (n = 25)
Change to palliative treatment	7.4% (n = 2)

Approximately three-quarters of patients (74%, n = 40) survived their ICU stay, with only one patient dying within the first 48 hours of admission. The standardised mortality ratio was 0.47. Following ICU discharge, 39 patients (72%) survived the hospital stay, and 33 (61%) remained alive at six-month follow-up. Overall, across the entire ICU cohort, 27 patients (50%) survived to one year after hospital discharge. In our cohort, one-year survival varied by transplant type: 7 of 9 patients with autologous transplants survived, whereas only 2 of 9 with allogeneic transplants survived. 

Among the one-year survivors, 93% (n = 25) had a good performance status, indicating maintenance of functional independence. These patients either remained on curative-intent treatment or under active surveillance, suggesting sustained functional status and disease control. Within this cohort, only one patient had documented relapsed disease. Two patients (7%) showed a marked decline in performance status (ECOG 4) and transitioned to palliative care.

## Discussion

Historically, HO patients were considered poor candidates for intensive care, primarily due to dismal survival rates [13]. Our results emphasise that HO patients can potentially benefit from ICU admission, demonstrating not only good survival but also maintenance of functional status. In this study of 54 critically ill patients with haematological malignancies, we observed a 61% survival rate at 6 months and a 50% survival rate at 12 months post-hospital discharge. Other encouraging findings include ongoing functional recovery, with most patients (93%) maintaining a good performance status at one-year follow-up, enabling continued cancer treatment as planned. These results highlight the potential for meaningful recovery and long-term benefit in HO patients admitted to intensive care.

The real-life setting of this study reflects the daily clinical challenges of managing HO patients. Our cohort showed good survival outcomes despite a moderate comorbidity burden (median Charlson Comorbidity Index: 3, P25-75 2-4) and high severity of illness at ICU admission, with 87% (n = 47) presenting with two or more organ dysfunctions, 33% requiring invasive mechanical ventilation, 61% vasoactive drugs, and 37% renal replacement therapy. Possible contributing factors include favourable patient characteristics at admission, good performance status, younger age, and a predominance of patients with either newly diagnosed haematological malignancies or relapsed disease eligible for life-spanning treatment. Our findings align with a study by Azoulay E et al., which reported a 43% one-year survival rate post-discharge in haematological patients admitted to intensive care between 2010 and 2011 [14]. Traditionally established prognostic factors for ICU survival, such as cancer-related characteristics, have little impact on the likelihood of ICU survival [15]. While the underlying disease influences long-term survival, evidence suggests that malignancy category alone should not preclude ICU admission [15]. Importantly, health status, as assessed by the ECOG performance status, and the burden of comorbidities are the main survival predictors [15]. Poor general condition one month before ICU admission [16], a high comorbidity burden, a prognosis of less than six months, or a condition with no life-spanning treatment is unlikely to derive benefit from ICU admission [15]. Our practice aligns with recent guidelines advocating full-code management in HO patients with good general condition, particularly those undergoing active treatment or in cancer remission [16].

Most patients in this cohort presented with substantial physiological compromise at the time of ICU admission, with 87% (n = 47) exhibiting two or more organ dysfunctions. The median time from hospital to ICU admission was 15.5 days, and 24% of patients required more than one referral before being accepted to the ICU. This delay may have contributed to the high burden of organ dysfunction at admission. Prior studies have shown that delayed ICU admission and cumulative organ injury are associated with higher mortality and reduced cancer treatment resumption [14,17]. The high rate of repeated referrals likely represents an opportunity to optimise ICU triage. Broader policies and a lower threshold for admitting HO patients may improve post-ICU survival and cancer treatment resumption by reducing accumulated organ dysfunction and frailty. Multidisciplinary collaboration between haematologists and intensivists could foster earlier recognition of organ failure and streamline ICU admissions.

While functional impairment and organ failures associated with critical care admission can delay or even render patients ineligible for resuming cancer treatment, the majority of survivors in this cohort (93%) not only returned to cancer care but also maintained a good performance status (ECOG 0 or 1). This reinforces the idea that ICU survival can pave the way for continued functional recovery and quality of life in a substantial subset of patients. This information is consistent with Azoulay E’s findings, where 80% of survivors with haematological malignancies admitted to ICU had no change in treatment intensity at 6 months post-intensive care [14]. Patients treated for haematological malignancies reported similar health-related quality of life whether or not they had received intensive care.

Haematology-specific considerations should also be taken into account. Cytopenias, for instance, increase infectious and haemorrhagic complications, adding a layer of difficulty and complexity to patient management. Our study demonstrates a favourable outcome despite a significant proportion of patients having severe neutropenia or undergoing haematopoietic stem-cell transplantation (HSCT). While neutropenia has been independently associated with poor outcomes [16], recent evidence suggests that persistent neutropenia during the ICU stay does not necessarily correlate with increased mortality [18]. As expected, in our cohort allogeneic HSCT with multiple organ failure carried a substantial risk of mortality, likely related to the underlying immunocompromised state. Allogeneic transplantation is a complex procedure undertaken with curative intent. Selected allogeneic HSCT recipients, especially those with controlled graft-versus-host disease (GVHD) and limited organ dysfunction, may derive the greatest benefit from ICU admission, and mechanical ventilation can be a viable intervention in this group [14]. Lastly, the median ICU length of stay was 8 days (P25-P75 3-15), aligning with findings that HO patients require longer ICU stays compared with the general population [5]. This extended time allows stabilisation, resolution of organ dysfunction, and re-evaluation of candidacy for cancer treatment.

Although the majority of patients in our study survived their ICU stay (74%), with only one death occurring within the first 48 hours, mortality continued to rise at the six-month follow-up. While many patients did not survive beyond this period, their ability to overcome an acute life-threatening event underscores the dual role of ICU survival: not only a critical milestone towards recovery but also an opportunity to ensure end-of-life care consistent with the patient’s wishes. These findings highlight the importance of early discussions about prognosis, quality of life, and goals of care to guide decisions throughout the clinical course.

Historically, perceptions of poor prognosis have led to restrictive ICU admission policies, which may inadvertently result in under-treatment and inequitable access to critical care. Conversely, aggressive interventions in patients with limited life expectancy or poor functional reserve may prolong suffering and divert resources from those most likely to benefit. Balancing the potential for meaningful recovery against the risk of therapeutic futility, while avoiding burdensome and inappropriate emergency decisions, requires collegiality and communication. Institutions should promote proactive communication between HO and critical care teams, establish clear admission criteria, and ensure palliative care is integrated early for patients with poor prognoses. Ultimately, the ethical goal is to align treatment intensity with the patient’s preferences and the realistic potential for recovery and quality of life.

Previous studies [6] included mixed cohorts of patients with solid cancers and haematological malignancies, whereas we focused on HO patients admitted to the ICU. Other strengths of this study include minimal loss to follow-up and the inclusion of follow-up details, such as functional status assessment and evaluation of continued cancer treatment following ICU discharge. Secondly, return to treatment and functional status were reported by the treating haematologist.

An important limitation of this study is the absence of standardised admission criteria for HO patients in our institution. This underscores the ethical and practical challenges surrounding ICU triage in patients with haematological malignancies. Other limitations include the small sample from a single hospital centre, the absence of a comparison group, and the retrospective, descriptive design. In the absence of multivariable modelling or a comparison group, the results reflect outcomes in a selected cohort from one centre. Large, multicentre prospective studies are needed to confirm these findings and to better define prognostic factors.

## Conclusions

This study of critically ill HO patients demonstrates a potential benefit from ICU admission, showing not only favourable survival rates but also ongoing functional recovery that enables continuation of planned cancer treatment. These findings support the value of ICU admission in selected critically ill patients with haematological malignancies and challenge longstanding assumptions that HO patients are poor candidates for intensive care.

Our results also highlight the need for institutional policies that promote early identification of high-risk patients and streamlined pathways for ICU referral. The observed mortality and functional decline within the first year in a subset of patients underscore the importance of timely discussions regarding patients’ wishes, goals of care, life expectancy, and preferences for life-sustaining treatments, particularly when ICU admission and deterioration are anticipated.

However, the absence of standardised admission criteria, the small sample derived from a single centre, the lack of a comparison group, and the retrospective nature of the study, along with reliance on descriptive analysis, limit the generalisability of these findings. Large, multicentre prospective studies are needed to confirm these results and better define prognostic factors.

## References

[1] Carmona-Bayonas A, Gordo F, Beato C, Castaño Pérez J, Jiménez-Fonseca P, Virizuela Echaburu J, Garnacho-Montero J (2018). Intensive care in cancer patients in the age of immunotherapy and molecular therapies: commitment of the SEOM-SEMICYUC. Med Intensiva (Engl Ed).

[2] Kroschinsky F, Stölzel F, von Bonin S, Beutel G, Kochanek M, Kiehl M, Schellongowski P (2017). New drugs, new toxicities: severe side effects of modern targeted and immunotherapy of cancer and their management. Crit Care.

[3] Tridente A, Dempsey NC, Khalifa M (2023). Predicting outcomes of hematological malignancy patients admitted to critical care. Front Hematol.

[4] Merz TM, Schär P, Bühlmann M, Takala J, Rothen HU (2008). Resource use and outcome in critically ill patients with hematological malignancy: a retrospective cohort study. Crit Care.

[5] Shrime MG, Ferket BS, Scott DJ (2016). Time-limited trials of intensive care for critically ill patients with cancer: How long is long enough?. JAMA Oncol.

[6] Atallah FC, Caruso P, Nassar AP Junior, Torelly AP, Amendola CP, Salluh JI, Romano TG (2023). High-value care for critically ill oncohematological patients: what do we know thus far?. Crit Care Sci.

[7] Hayden PJ, Roddie C, Bader P (2022). Management of adults and children receiving CAR T-cell therapy: 2021 best practice recommendations of the European Society for Blood and Marrow Transplantation (EBMT) and the Joint Accreditation Committee of ISCT and EBMT (JACIE) and the European Haematology Association (EHA). Ann Oncol.

[8] Azoulay E, Timsit JF, Sprung CL (2009). Prevalence and factors of intensive care unit conflicts: the conflicus study. Am J Respir Crit Care Med.

[9] Oken MM, Creech RH, Tormey DC, Horton J, Davis TE, McFadden ET, Carbone PP (1982). Toxicity and response criteria of the Eastern Cooperative Oncology Group. Am J Clin Oncol.

[10] Singer M, Deutschman CS, Seymour CW (2016). The Third International Consensus Definitions for sepsis and septic shock (Sepsis-3). JAMA.

[11] Döhner H, Wei AH, Appelbaum FR (2022). Diagnosis and management of AML in adults: 2022 recommendations from an international expert panel on behalf of the ELN. Blood.

[12] von Elm E, Altman DG, Egger M, Pocock SJ, Gøtzsche PC, Vandenbroucke JP (2014). The Strengthening the Reporting of Observational Studies in Epidemiology (STROBE) Statement: guidelines for reporting observational studies. Int J Surg.

[13] Lloyd-Thomas AR, Wright I, Lister TA, Hinds CJ (1988). Prognosis of patients receiving intensive care for lifethreatening medical complications of haematological malignancy. Br Med J (Clin Res Ed).

[14] Azoulay E, Mokart D, Pène F (2013). Outcomes of critically ill patients with hematologic malignancies: prospective multicenter data from France and Belgium--a groupe de recherche respiratoire en réanimation onco-hématologique study. J Clin Oncol.

[15] Cheng Q, Tang Y, Yang Q, Wang E, Liu J, Li X (2016). The prognostic factors for patients with hematological malignancies admitted to the intensive care unit. Springerplus.

[16] Meert AP, Wittnebel S, Holbrechts S (2021). Critically ill cancer patient's resuscitation: a Belgian/French societies' consensus conference. Intensive Care Med.

[17] Orvain C, Beloncle F, Hamel JF (2018). Allogeneic stem cell transplantation recipients requiring intensive care: time is of the essence. Ann Hematol.

[18] van Vliet M, van den Boogaard M, Donnelly JP, Evers AW, Blijlevens NM, Pickkers P (2014). Long-term health related quality of life following intensive care during treatment for haematological malignancies. PLoS One.

